# Effect of Electrolytic-Plasma Hardening Parameters on the Microstructure and Mechanical Behavior of 20X Steel

**DOI:** 10.3390/ma19183907

**Published:** 2026-09-14

**Authors:** Arystanbek Kussainov, Zarina Aringozhina, Gulnara Zhunissova, Lyaila Bayatanova, Bauyrzhan Rakhadilov, Moldir Kaliaskarova

**Affiliations:** 1 International School of Engineering, D. Serikbayev East Kazakhstan Technical University, Ust-Kamenogorsk 070010, Kazakhstan; arys20055@gmail.com (A.K.); zaringozhina@edu.ektu.kz (Z.A.); leila_1809@mail.ru (L.B.); mkaliaskarova@edu.ektu.kz (M.K.); 2 PlasmaScience LLP, Ust-Kamenogorsk 070000, Kazakhstan; rakhadilovb@gmail.com

**Keywords:** electrolytic-plasma hardening, 20X steel, surface hardening, microstructure, phase composition, microhardness, tribological properties, surface melting

## Abstract

This study investigates the effects of applied voltage and treatment duration during electrolytic-plasma hardening (EPH) on the microstructure, phase composition, hardness, surface condition, and tribological behavior of 20X steel. EPH was performed in a 12 wt.% Na_2_CO_3_ aqueous electrolyte at voltages of 280, 300, and 320 V for 4 and 6 s. Scanning electron microscopy, energy-dispersive X-ray spectroscopy, X-ray diffraction, instrumented indentation, surface profilometry, and ball-on-disk tribological testing were used to characterize the treated specimens. The 280 V/4 s, 300 V/4 s, 320 V/4 s, and 280 V/6 s conditions produced thermally modified surface layers without visible surface damage, whereas treatment at 300 and 320 V for 6 s resulted in localized surface melting. Among the undamaged specimens, the highest hardness was obtained at 280 V, reaching 329.6 ± 15.8 HV after 4 s and 332.1 ± 26.3 HV after 6 s, corresponding to an approximately 1.83–1.85-fold increase relative to the initial value of 180 HV. XRD revealed a predominantly α-Fe-based matrix, while weak secondary reflections could not be assigned reliably to specific phases. The minimum steady-state coefficient of friction was obtained at 300 V/4 s (0.444 ± 0.054), whereas the narrowest wear track was observed at 320 V/4 s (379.48 ± 36.24 μm). No direct correlation was found between hardness and tribological response, indicating that surface roughness and microstructural state should also be considered when selecting treatment parameters. The results define a stable EPH processing window for 20X steel and demonstrate that parameter selection should be based on a combined assessment of surface integrity, hardness, and tribological performance.

## 1. Introduction

Increasing the hardness and wear resistance of machine-part surfaces is one of the key challenges in modern mechanical engineering, especially for components operating under contact loads, abrasive wear, and fatigue conditions, such as oil and gas equipment, pipeline valves, gears, shafts, and friction-pair components [1]. Conventional quenching is a widely used heat-treatment process in which steel is heated to a temperature within the austenitizing range, held for a sufficient time to obtain an austenitic structure, and subsequently cooled rapidly in an appropriate quenching medium, such as water or oil. The rapid cooling promotes the transformation of austenite into martensite, thereby increasing the hardness and strength of the steel [1,2]. For low-carbon and low-alloy steels requiring surface hardening, carburizing is widely used to enrich the surface layer with carbon. Depending on the required properties and the selected heat-treatment route, carburizing may be followed by quenching to promote martensitic transformation and increase surface hardness. For 20X steel in particular, conventional surface strengthening may involve carburizing followed by quenching, resulting in a hardened surface layer while retaining a relatively tough core [2]. However, such treatment is characterized by long processing times, high energy consumption, and prolonged furnace heating [2]. Other widely used surface-hardening methods include induction, laser, and plasma hardening, ion nitriding, and various chemical–thermal treatments [3,4,5]. Despite their effectiveness, many of these methods require expensive equipment, complex surface preparation, or lengthy processing cycles, which can limit their application in small-batch and repair production [3]. Electrolytic-plasma hardening (EPH) is considered a promising alternative to traditional surface hardening methods due to its high processing speed, relatively low energy consumption, and the absence of the need for vacuum equipment. The process is carried out using a standard direct current source and an aqueous electrolyte based on readily available salts, and the treatment duration is only a few seconds [3,4,5]. Furthermore, the localized nature of heating enables selective hardening of the working surface while minimizing structural changes in the core, which is particularly important for components subjected to bending and torsion. An additional advantage of the method is that rapid cooling of the heated surface occurs directly in the circulating electrolyte. This rapid cooling represents the quenching stage of the overall electrolytic-plasma hardening process and eliminates the need to transfer the part to a separate quenching medium [4,6].

The mechanism of EPH is associated with the formation of a vapor-gas envelope near the cathode surface when a sufficiently high DC voltage is applied between the workpiece and the electrolyte [7,8]. Electrical discharges developing within this envelope generate intense localized heating of the steel surface. Depending on the processing parameters, the surface temperature may reach the austenitizing range, enabling phase transformation in the near-surface region [2,6,7]. After the voltage is switched off, the heated surface is rapidly cooled by the circulating electrolyte. This quenching stage promotes the transformation of austenite into martensite and the formation of a hardened surface layer [2,6,7]. Previous studies on 20X (20Ch) steel have also reported changes in the elemental composition of the surface layer during electrolytic-plasma processing, which may be associated with interactions between the electrolyte, electrode materials, and the treated surface [2]. Such compositional changes may additionally influence the resulting mechanical and tribological behavior.

The material selected for this study is 20X steel (GOST 4543-2016 [9], GOST 33260-2015 [10]; foreign equivalent AISI 5120), a low-alloy chromium case-hardenable steel widely used for manufacturing components operating under contact and cyclic loads: gear wheels, gear shafts, pins, pipeline valve components, and sucker rod pairs for oil and gas equipment [11,12,13]. 20X steel combines good machinability, high core toughness, and the potential for effective surface hardening, making it widely used both in industry and in research into various methods of thermal and chemothermal treatment, including electrolytic-plasma hardening [5,6]. This makes it a convenient model material for studying the mechanisms governing the formation of a hardened surface layer. It has previously been shown that electrolytic-plasma hardening promotes the formation of a martensitic structure, increases the depth of the hardened layer, and significantly increases the surface hardness of various structural steels [4,5,6,7,8,14,15]. In particular, Sagdoldina et al. [16] observed the formation of acicular martensite during the treatment of 40X steel, whereas Kombayev et al. [2,11] demonstrated an increase in hardness and a rise in the content of alloying elements in the surface layer of 20X steel following cyclic treatment. A generalization of the patterns governing the formation of the hardened layer under various EPH conditions is presented in the review by Belkin and Kusmanov [3,4,5,6]. The performance properties of hardened surfaces are determined not only by hardness but also by tribological behavior, including the coefficient of friction and wear resistance during contact interaction. Magazov et al. [17] investigated the effect of ultrasonic nanocrystalline surface modification and electrolytic-plasma treatment on the properties of AISI 52100 steel and demonstrated the relationship between hardness, coefficient of friction, and wear volume. However, most studies on electrolytic-plasma hardening of 20X steel have focused primarily on the analysis of microstructure and hardness, whereas the comprehensive relationship between EPH parameters, structural-phase state, and tribological characteristics has not been sufficiently investigated. Of particular practical interest is determining the transition boundary from stable surface hardening to local surface melting as the applied voltage and treatment duration increase. Establishing this boundary is necessary for selecting technologically acceptable EPH parameters and preventing surface damage.

The novelty of the present study lies in the systematic evaluation of the combined effects of applied voltage and treatment duration on the microstructure, elemental and phase composition, hardness, and tribological behavior of 20X steel, with particular emphasis on identifying the transition between stable surface hardening and local surface melting. The contribution of this work is the determination of technologically acceptable EPH parameters based on the combined assessment of surface integrity, hardness, and tribological performance.

The objective of this study was to investigate the effects of applied voltage (280, 300, and 320 V) and treatment duration (4 and 6 s) during electrolytic-plasma hardening on the microstructure, elemental and phase composition, microhardness, and tribological characteristics of 20X steel, as well as to determine the conditions for stable hardening and the conditions under which local surface melting occurs.

## 2. Materials and Methods

The subject of the study was 20X alloy structural steel, the chemical composition of which is shown in Table 1. The specimens were fabricated as rectangular plates measuring 30 × 25 × 20 mm. Surface preparation was performed by sequential manual grinding using abrasive paper with grit sizes ranging from P100 to P2500. The initial microhardness of the steel, measured prior to electrolytic-plasma treatment, was 180 HV.

**Figure 1:**
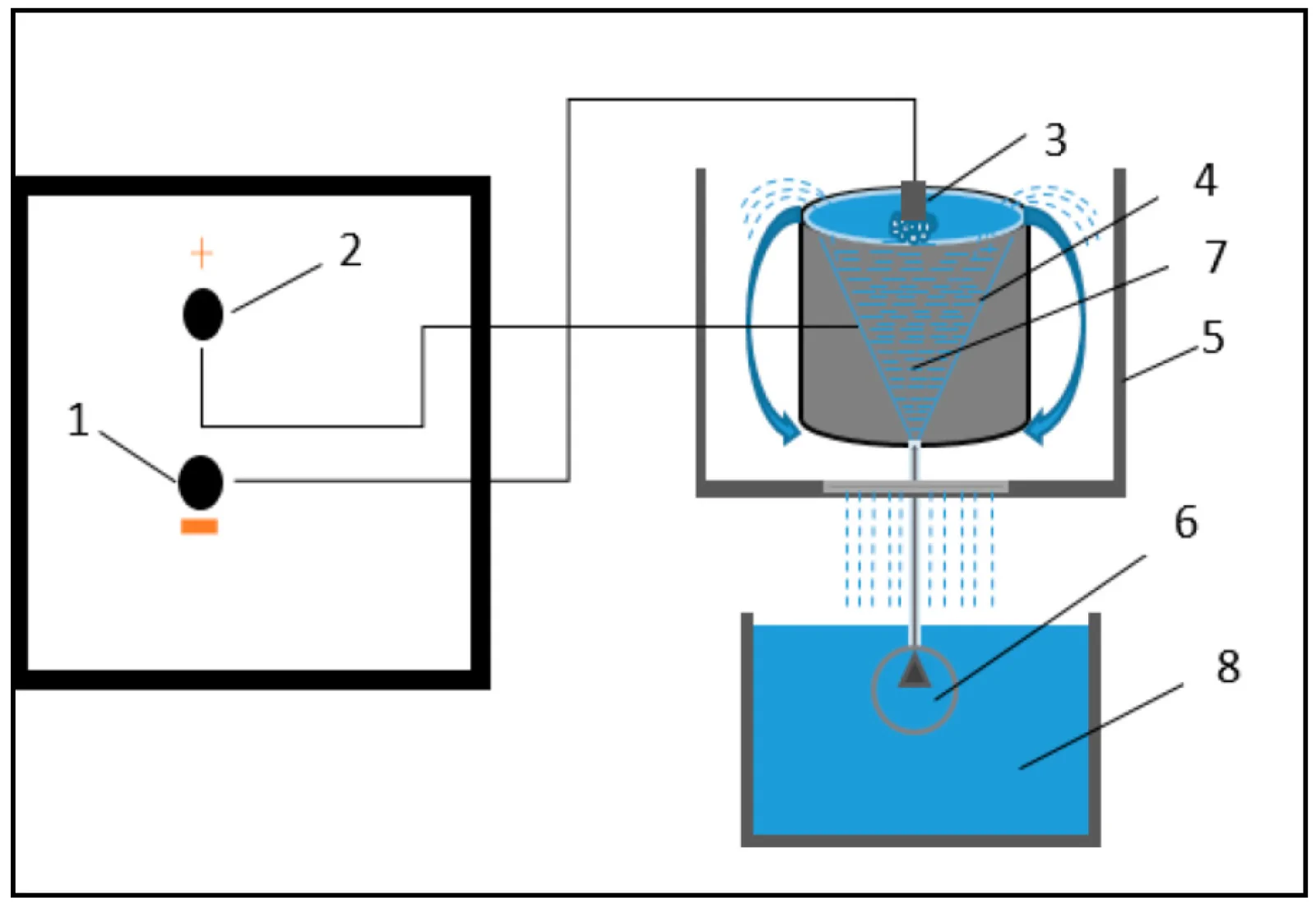
Schematic of the electrolytic-plasma hardening setup: 1—anode; 2—cathode; 3—sample under processing; 4—conical stainless steel electrolytic cell; 5—settling tank; 6—pump; 7—electrolyte; 8—electrolyte bath.

Electrolytic-plasma hardening was performed on a laboratory setup, the diagram of which is shown in Figure 1.

The setup operates as follows. The pump (6) circulates the electrolyte (7) from the electrolytic bath (8) into the conical cell (4), which is made of stainless steel and connected as the anode (1). The sample (3) to be treated, secured to a current-carrying rod and connected as the cathode (2), is immersed in the circulating electrolyte flow. The cathodic polarity was selected because the present treatment is based on cathodic electrolytic-plasma hardening. In this configuration, the steel specimen is connected to the negative terminal and acts as the cathode, whereas the stainless-steel electrolytic cell is connected to the positive terminal and serves as the anode. Under sufficiently high applied voltage, a vapor-gas envelope forms near the cathode surface, and electrical discharges developing within this envelope provide intense localized heating of the treated surface [6,8]. This cathodic configuration is commonly used for electrolytic-plasma hardening of steels and enables rapid localized surface heating followed by rapid cooling in the circulating electrolyte after the voltage is switched off [6,8]. This rapid cooling constitutes the quenching stage of the overall electrolytic-plasma hardening process. The spent electrolyte flows into a settling tank (5) and is then returned to the circulation system.

According to Kombayev et al. [2], the austenitizing temperature required for phase transformation of 20X (20Ch) steel is approximately 840–860 °C. During electrolytic-plasma hardening, rapid localized heating can bring the near-surface region of the steel into this temperature range within several seconds. Literature data indicate that cathodic plasma-electrolytic treatment may involve heating rates of approximately 10–200 °C/s [18]. For related low-carbon steels subjected to EPH, cooling rates of approximately 12–80 °C/s in the region of minimum austenite stability and 20–140 °C/s in the martensitic transformation range have been reported [18]. In the present study, the surface temperature and cooling rate were not measured directly; therefore, these literature values are provided only to characterize the expected thermal conditions of the EPH process. The actual thermal cycle may vary depending on the applied voltage, specimen geometry, electrolyte temperature, and electrolyte flow conditions.

An aqueous solution of sodium carbonate (Na_2_CO_3_) with a concentration of 12 wt.% (88 wt.% water) was used as the electrolyte. Electrolytic-plasma hardening (EPH) was performed at voltages of 280, 300, and 320 V with treatment durations of 4 and 6 s, corresponding to six experimental conditions (Table 2). For each experimental condition, the current in the electrical circuit was recorded as a response parameter of the EPH process.

The current was not an independently controlled processing parameter but represented the electrical response of the electrolyte–sample system under each treatment condition. The recorded current values did not exhibit a monotonic dependence on either applied voltage or treatment duration. Since time-resolved current and voltage signals were not acquired in the present study, the origin of these differences cannot be determined directly. Previous studies have shown that the formation and disruption of the vapor-gas envelope may influence the electrical resistance of the near-cathode region and cause fluctuations in discharge current [7,8]. However, this mechanism was not directly verified in the present experiments. Therefore, the current values presented in Table 2 should be regarded only as characteristics of the electrical response under each EPH condition rather than as independent indicators of the intensity or stability of the treatment process.

The microstructure of the cross-section, the morphology of the wear tracks, the elemental composition, and the distribution of chemical elements across the depth of the surface layer were examined using a TESCAN VEGA4 LMH scanning electron microscope (TESCAN, Brno, Czech Republic) equipped with an energy-dispersive X-ray spectrometer (EDS). The investigations were conducted in the secondary electron (SE) mode at magnifications of ×500 and ×2000. To reveal the microstructure, cross-sections were etched with a solution of nitric acid (4 wt.%) in ethyl alcohol (96 wt.%), which enabled visualization of the microstructural contrast, grain boundaries, and characteristic morphological features of the thermally modified regions.

X-ray diffraction (XRD) analysis of the surface layer was performed on a DW-XRD-27MINI diffractometer using Cu Kα radiation (λ = 1.5406 Å) in the 2θ range of 20–90°, with a scan step of 0.02°, with an exposure time of 0.5 s per step.

The hardness and indentation modulus (EIT) of the cross-sections were determined using a FISCHERSCOPE HM2000 measuring system (Sindelfingen, Germany) by instrumented indentation. EIT represents the indentation-derived modulus determined from the unloading portion of the load–displacement curve and should be distinguished from the conventional Young’s modulus, E. The tests were performed on cross-sectional polished sections of the samples. The load was gradually increased to 300 mN over 20 s, followed by a 5-s hold at the maximum load.

Tribological studies were conducted on an Anton Paar TRb3 tribometer (Anton Paar GmbH, Graz, Austria) using the “ball-on-disc” configuration, which complies with the international standards ASTM G99-23 [19]. The tests were conducted at room temperature without lubricants; a 3-mm-radius ball made of 100Cr6 material was used as the counterbody. The tribological tests were conducted under a load of 6 N at a linear speed of 3 cm/s, with a wear radius of 3 mm and a total wear length of 40 m.

## 3. Results

### 3.1. Microstructure and Elemental Composition of the Surface Layer

**Figure 2:**
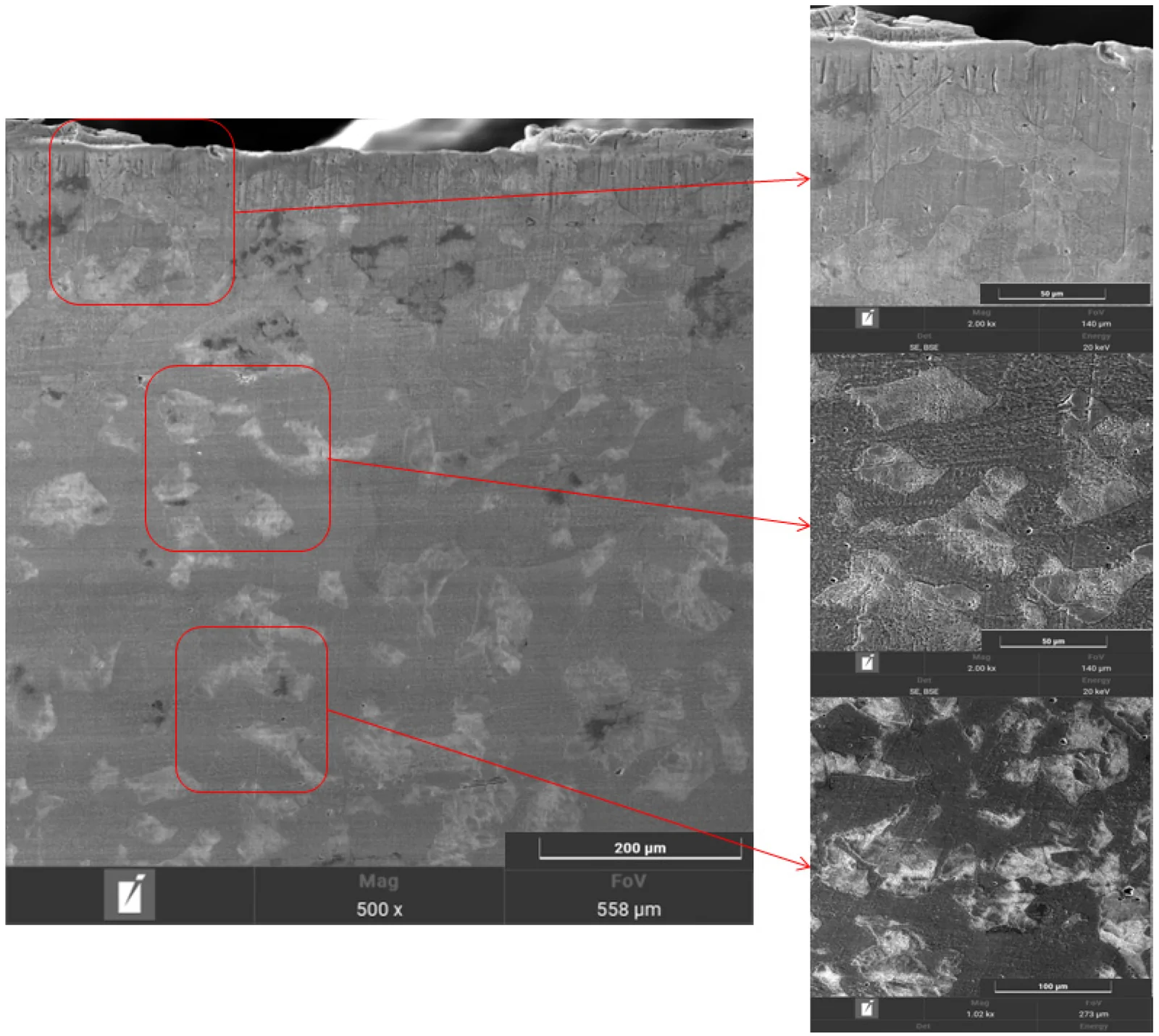
Microstructure of a cross-section of Sample 1.

**Figure 3:**
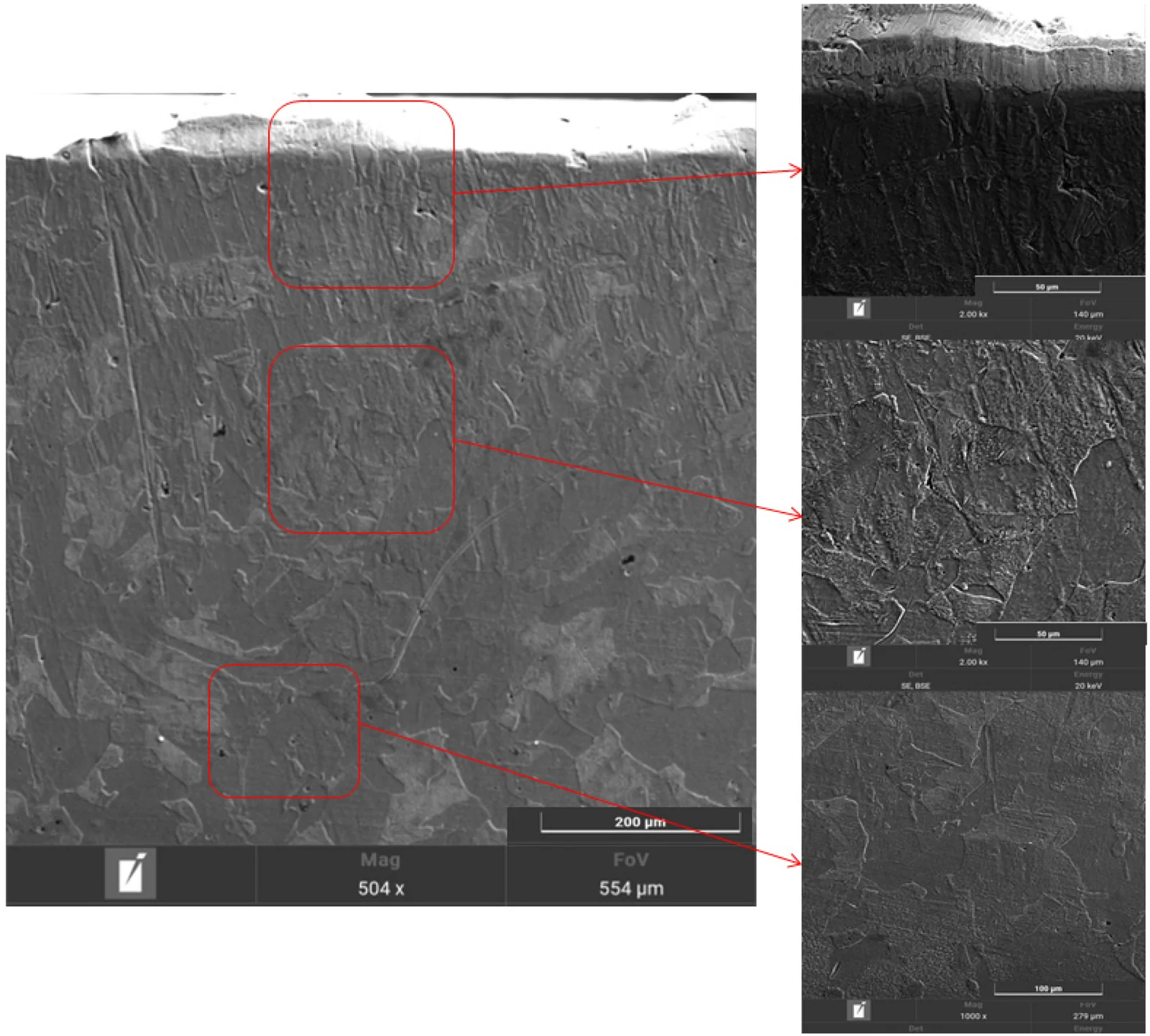
Cross-sectional microstructure of sample 2.

**Figure 4:**
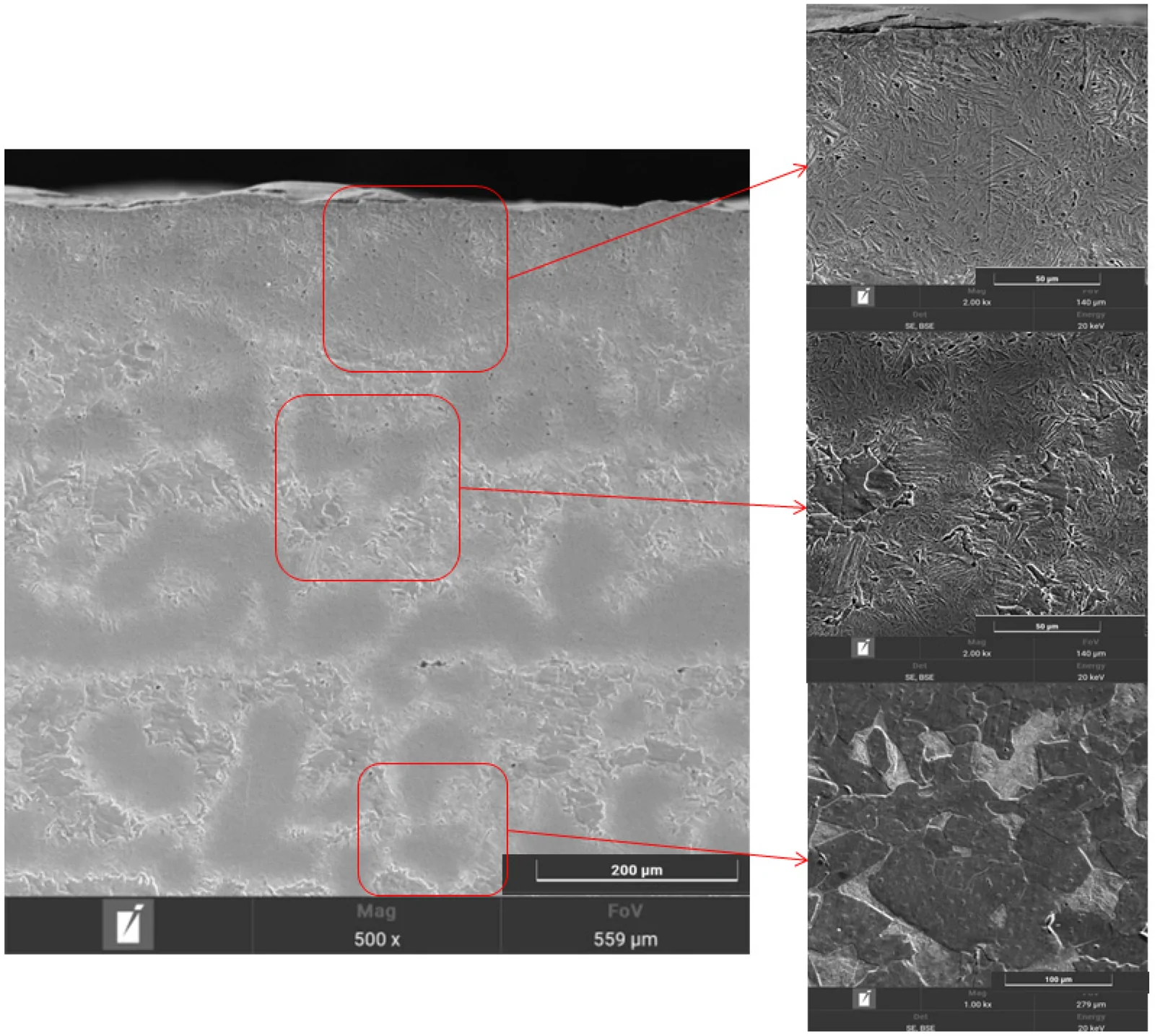
Cross-sectional microstructure of sample 3.

**Figure 5:**
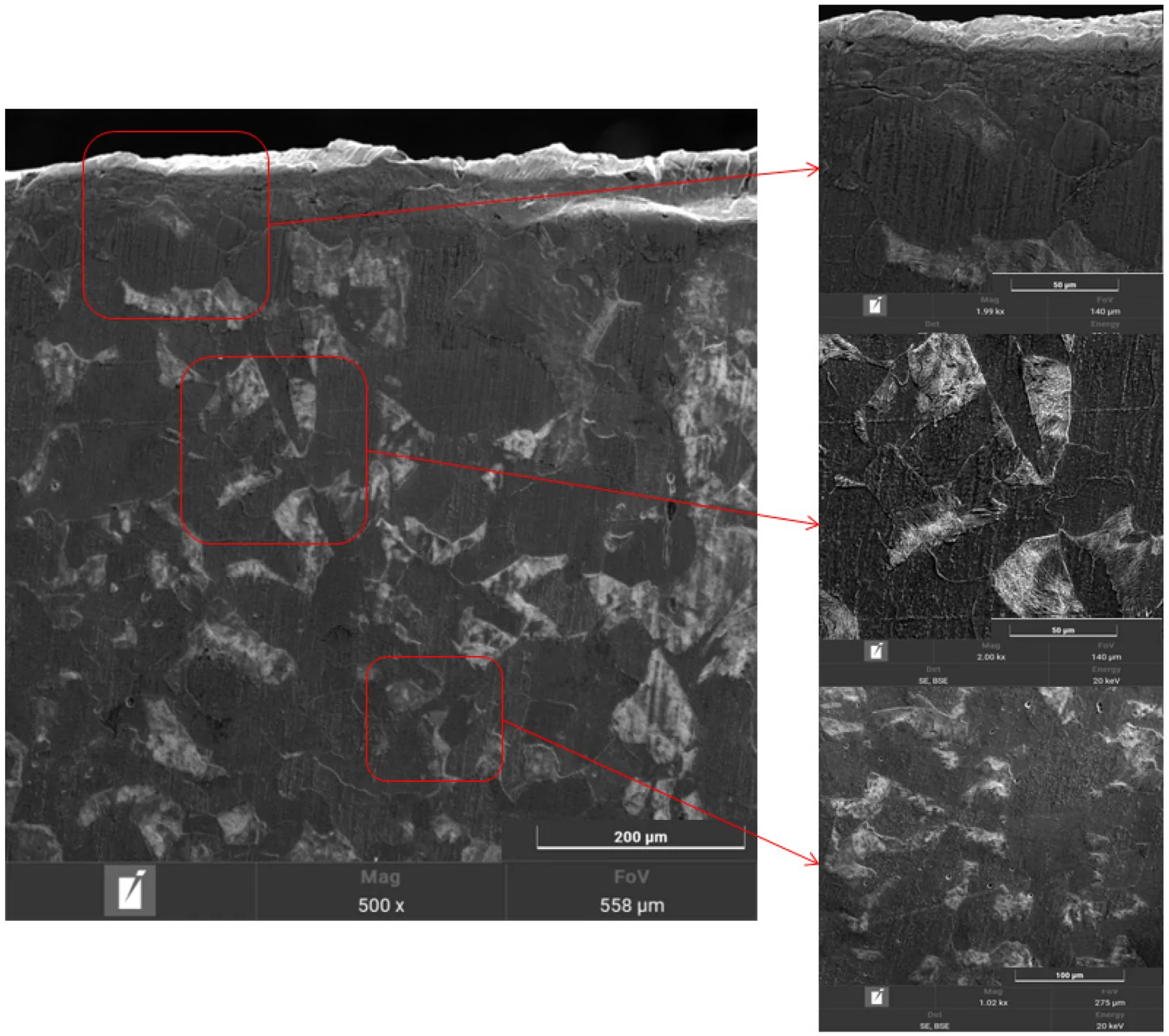
Cross-sectional microstructure of sample 4.

**Figure 6:**
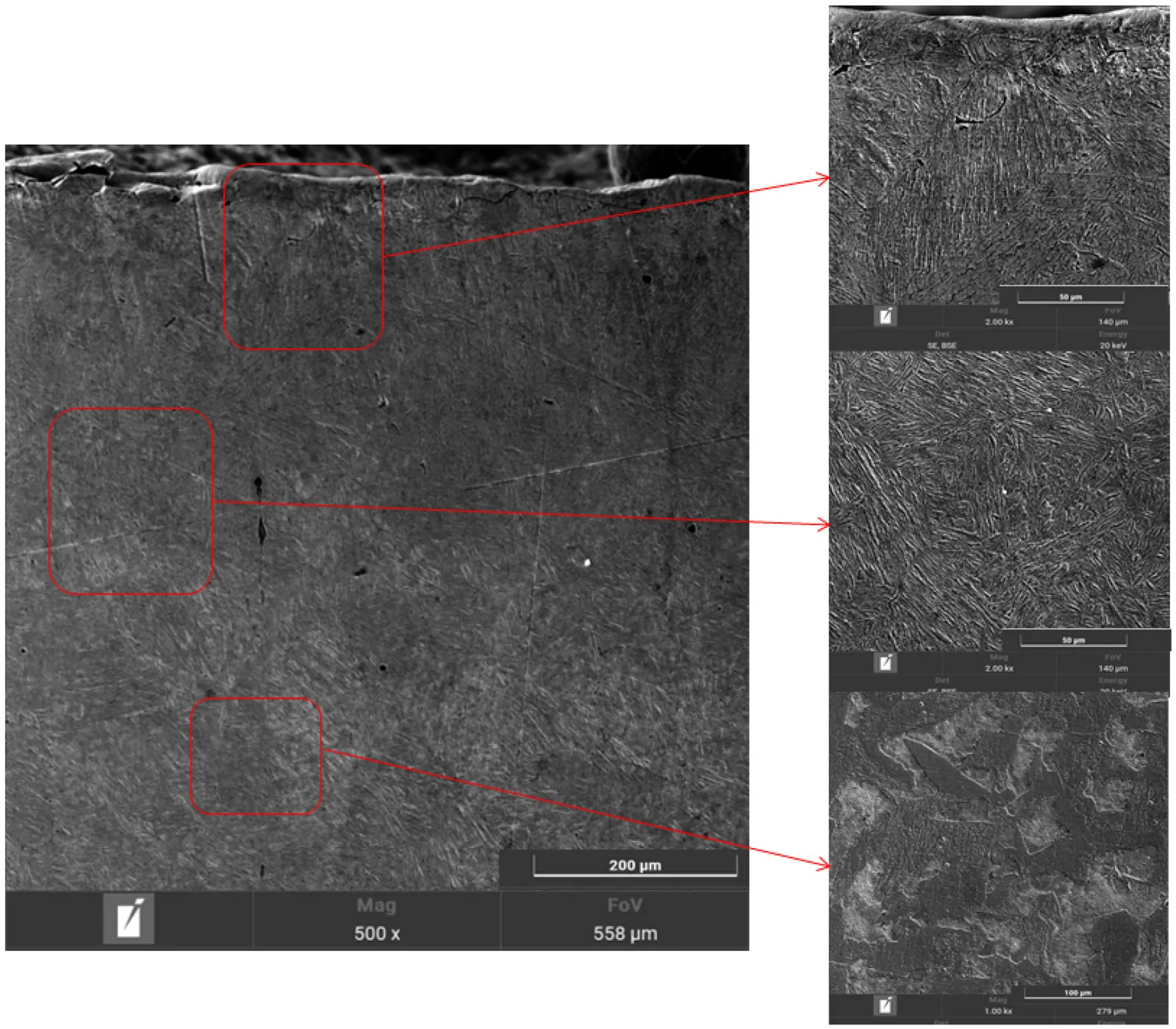
Microstructure of a cross-section of sample 5.

**Figure 7:**
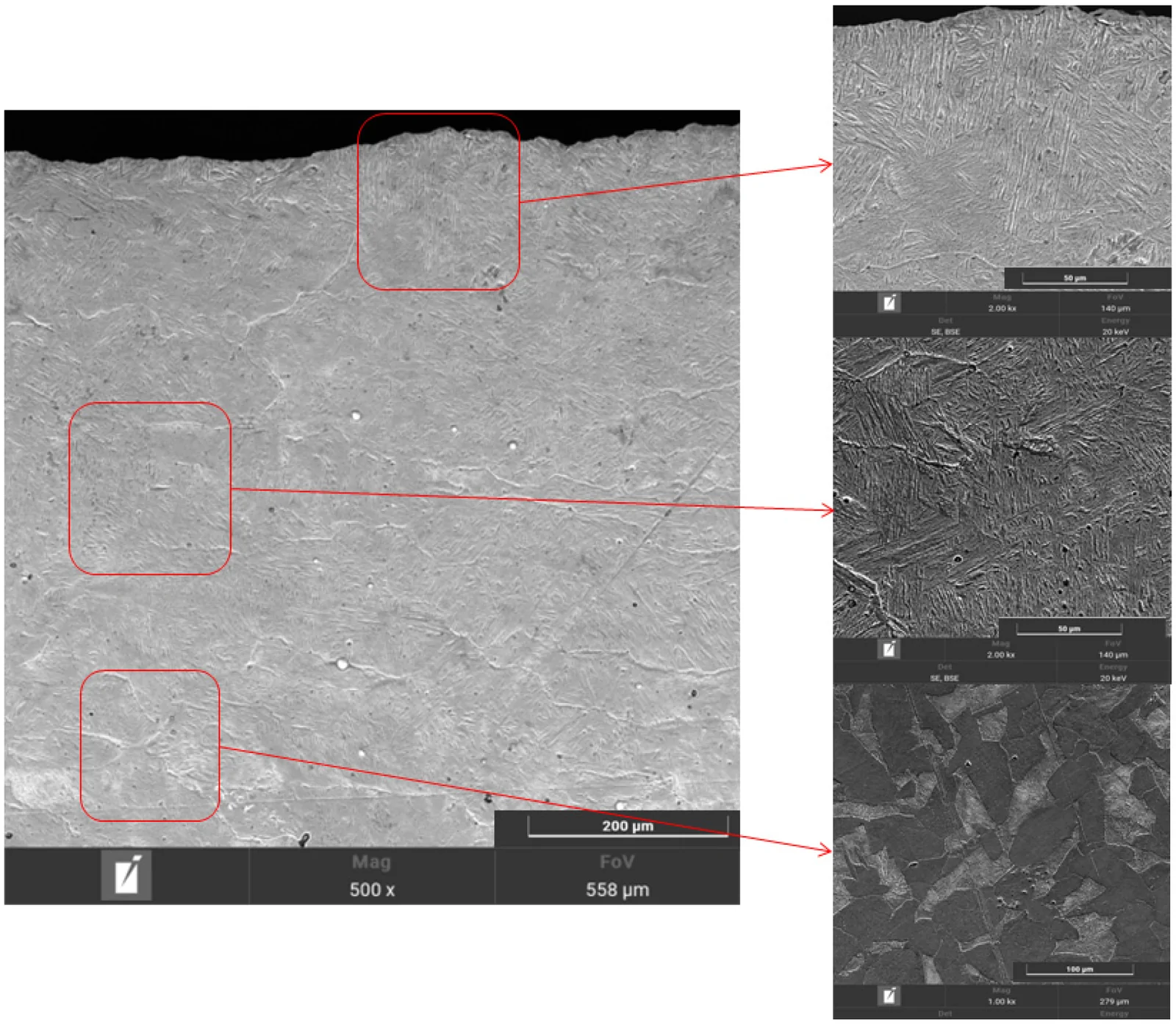
Microstructure of a cross-section of sample 6.

After electrolytic-plasma hardening, the specimens treated at 280 V/4 s, 300 V/4 s, 320 V/4 s, and 280 V/6 s retained their surface integrity. Localized melting was observed at 300 V/6 s and 320 V/6 s. The melted samples were excluded from tribological testing but were used for microstructural, elemental, and phase analysis. The microstructures of the cross-sections of all studied samples are shown in Figure 2, Figure 3, Figure 4, Figure 5, Figure 6 and Figure 7. At the 280 V/4 s condition, a thermally altered surface layer formed with a clearly defined transition boundary to the base metal. The average layer thickness, determined from the results of three measurements, was 61.51 ± 9.92 μm.

As can be seen in Figure 2, the formed layer exhibits a heterogeneous structure with patchy contrast and a weakly expressed needle-like morphology. Individual structural regions are distinguishable; however, the characteristic needle-like substructure is significantly less pronounced than in the sample treated at 280 V/6 s (Figure 5). This indicates a less pronounced structural rearrangement of the surface layer at a treatment duration of 4 s.

The microstructure of the sample treated at 300 V/4 s is shown in Figure 3. In the middle and lower parts of the thermally altered layer, a heterogeneous structure with spotty contrast and weakly expressed needle-like morphology is observed, generally similar to the structure of the 280 V/4 s sample. In the near-surface region, a thin light-colored band is visible, immediately adjacent to the surface. The change in contrast in this region may be related to the characteristics of the formed structure or to varying etching intensities; however, further investigation is needed to unambiguously determine its nature. The microstructure of the sample treated at 320 V/4 s is shown in Figure 4. In the near-surface and central parts of the thermally altered layer, a more distinctly expressed needle-like substructure is observed than in the 280 V/4 s and 300 V/4 s samples.

As can be seen in Figure 4, a distinctly needle-like substructure is observed in the near-surface and central regions of the thermally altered layer. Near the surface, fine, predominantly parallel-oriented structures form, whereas in the middle part of the layer, they acquire a more pronounced fan-shaped orientation. This morphology may be associated with structural transformations occurring during rapid heating and subsequent cooling of the surface. In the lower part of the layer, near the boundary with the base metal, larger polygonal regions with clearly defined boundaries and a less pronounced internal substructure predominate. This indicates a gradual decrease in the intensity of structural changes with increasing distance from the treated surface.

The microstructure of the sample treated at 280 V/6 s is shown in Figure 5. Clearly distinguishable polygonal regions are observed throughout the thickness of the thermally altered layer, most pronounced in the near-surface region. Within individual regions, a oriented needle-like substructure is visible, which becomes less pronounced in the middle and lower parts of the layer. Compared to the 280 V/4 s sample (Figure 2), the structure is characterized by more distinct boundaries between individual regions and a more pronounced internal substructure. The observed changes indicate a more intense structural rearrangement of the surface layer as the treatment duration is increased to 6 s. The microstructure of the sample treated at 300 V/6 s and subjected to localized surface melting is shown in Figure 6. In the near-surface and middle regions of the thermally altered layer, a dense, fine-grained needle-like substructure is observed, the morphology of which changes only slightly with depth. The formation of such a structure may be associated with intense thermal exposure and subsequent rapid cooling of the surface. In the lower part of the examined zone, instead of the oriented needle-like morphology, larger, irregularly shaped light-colored regions without a pronounced predominant orientation are observed. In contrast to undamaged specimens, a clear boundary between the thermally altered layer and the base metal is practically absent, indicating a deeper penetration of structural changes under these conditions.

Figure 7 shows a cross-section of a specimen treated at 320 V/6 s. As a result of the treatment, the specimen’s surface underwent localized melting. In the upper and central parts of the affected zone, a fine-grained, needle-like morphology is observed, similar to that seen under the 300 V/6 s treatment (Figure 6). However, as one approaches the base metal, the structure changes noticeably: large, angular regions with predominantly straight boundaries form. Such a morphology was not observed under the other tested conditions and indicates a different nature of structural rearrangement as the voltage increases to 320 V. The transition from the altered region to the core of the specimen is weakly defined, indicating that the thermal effect extends to a considerable depth.

**Figure 8:**
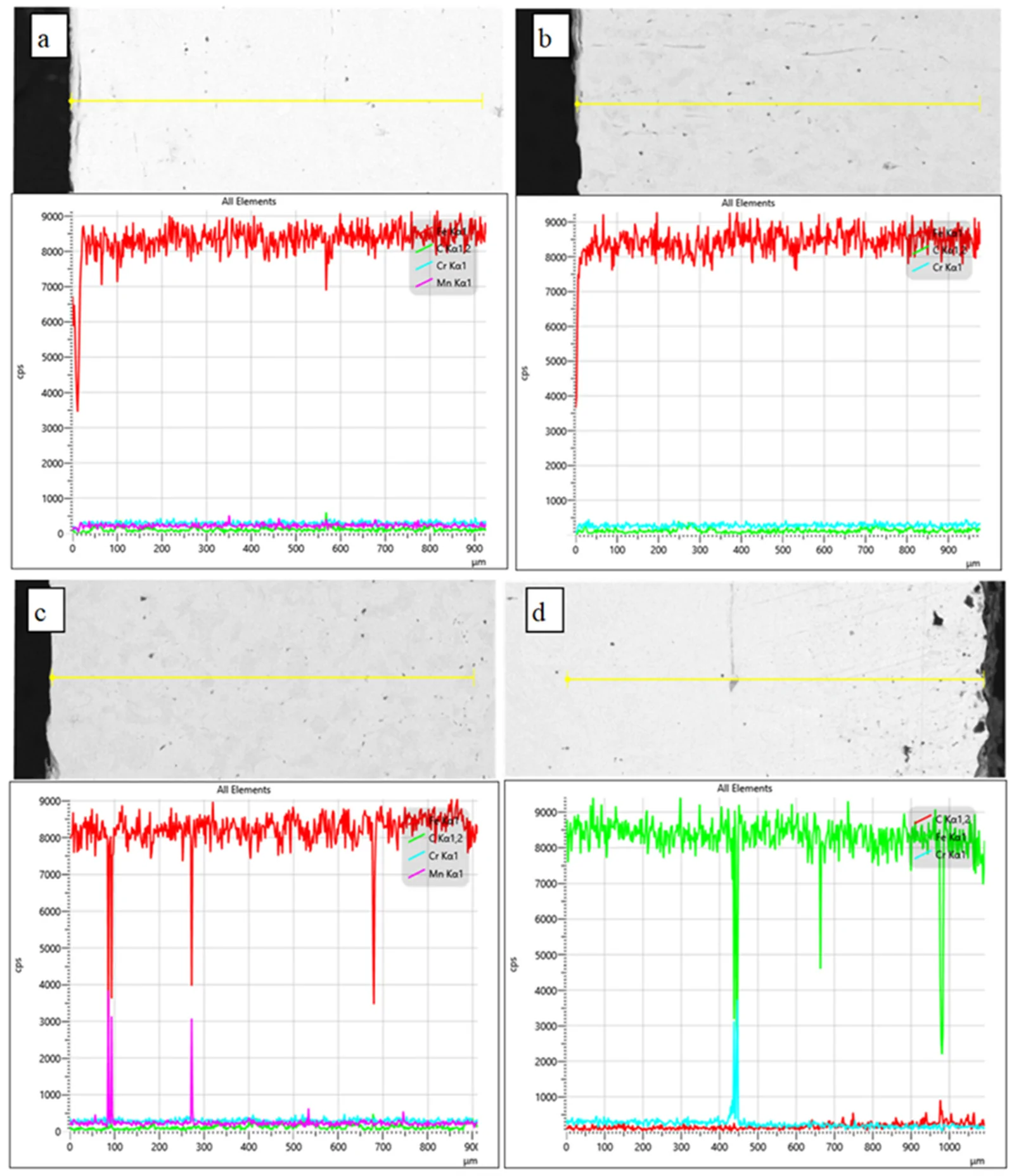

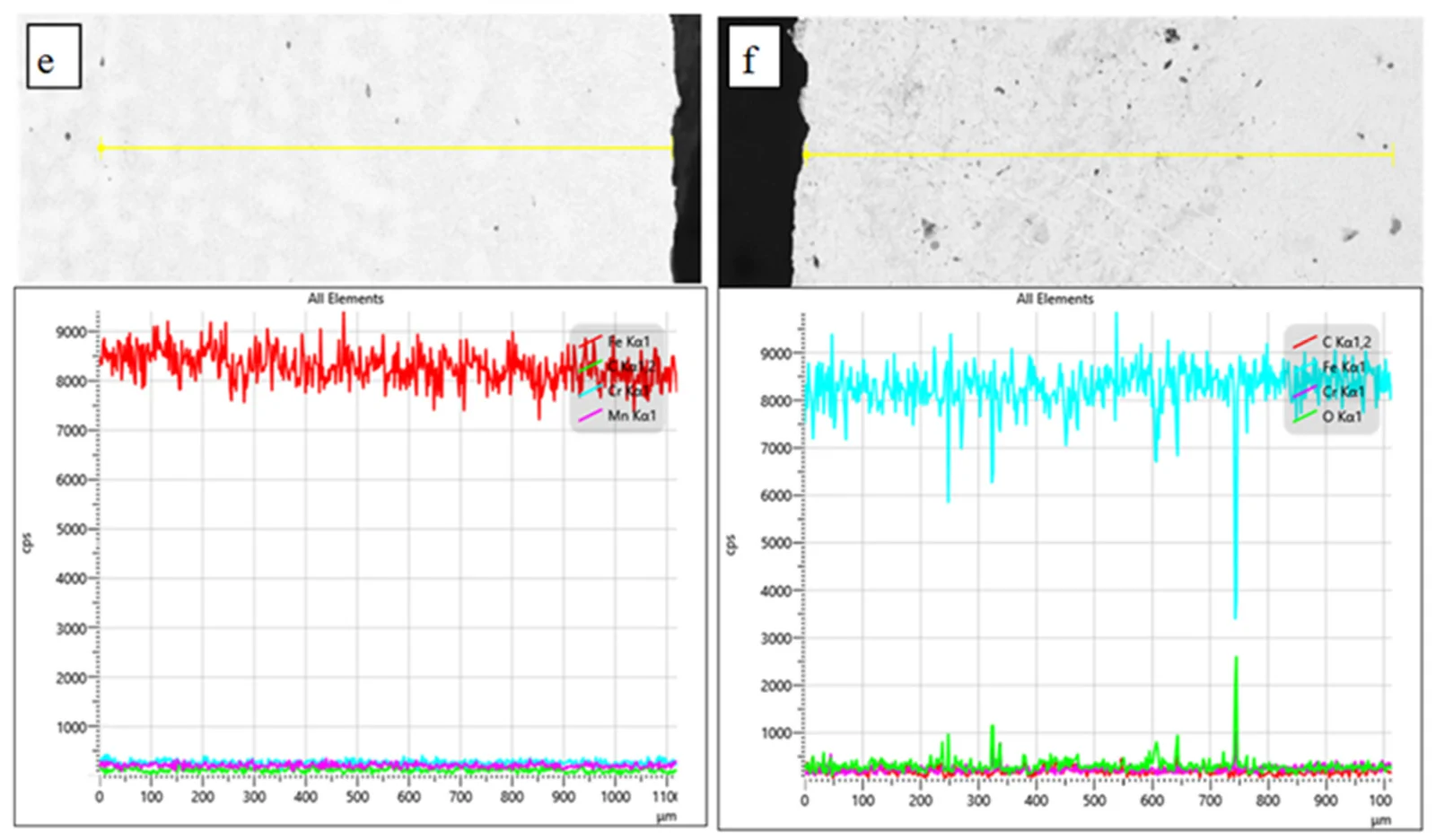
Results of linear EDS line scanning of the samples: (**a**) 280 V, 4 s; (**b**) 300 V, 4 s; (**c**) 320 V, 4 s; (**d**) 280 V, 6 s; (**e**) 300 V, 6 s; (**f**) 320 V, 6 s.

A comparison of the microstructures shown in Figure 2, Figure 3, Figure 4, Figure 5, Figure 6 and Figure 7 reveals that the nature of the changes in the surface layer is determined by a combination of voltage and treatment duration. At the treatment conditions of 280 V/4 s, 300 V/4 s, 320 V/4 s, and 280 V/6 s, a localized thermally altered layer forms with a distinct transition boundary to the base metal. An increase in voltage at a treatment duration of 4 s is accompanied by a gradual intensification of the needle-like morphology, which is most pronounced at 320 V/4 s. Increasing the treatment time to 6 s at 280 V leads to a more distinct manifestation of polygonal regions and the internal substructure without compromising the surface integrity. At 300 and 320 V for 6 s, localized melting, expansion of the thermally altered zone, and weakening of its transition boundary to the base metal are observed. Thus, the combination of increased voltage and a treatment duration of 6 s leads to conditions beyond the stable electrolytic-plasma hardening regime. To correlate the identified structural changes with the characteristics of the elemental composition, linear EDA scanning was performed on the cross-sections of all tested specimens. The scanning direction was set from the treated surface toward the base metal. The elemental distribution profiles are shown in Figure 8, and the results of the semi-quantitative analysis are presented in Table 3.

According to Figure 8 and Table 3, samples with an undamaged surface are characterized by relatively similar values of the recorded elemental composition. The Fe content ranged from 88.77 to 90.30 wt.%, C from 7.70 to 9.10 wt.%, Cr—0.84–0.90 wt%. Oxygen was not detected under the 280 V/4 s, 300 V/4 s, and 280 V/6 s conditions, whereas after treatment at 320 V/4 s, its content was 0.99 wt%. In the samples subjected to melting, a decrease in the Fe content was observed to 86.20 wt.% at 300 V/6 s and to 84.04 wt.% at 320 V/6 s. At the same time, the C signal increased to 11.23 and 13.07 wt.%, and the O signal to 1.62 and 2.33 wt.%, respectively. The Cr content decreased to 0.65 and 0.57 wt.%. The most pronounced changes were recorded at the 320 V/6 s condition. The increase in the oxygen signal may be associated with the intensification of oxidative processes as the voltage and treatment duration increase. At the same time, carbon values should be interpreted with caution due to the limited accuracy of the EDS in the quantitative determination of light elements. Therefore, the obtained data reflect comparative trends in changes in elemental composition but do not allow for the unambiguous identification of the formation of specific chemical compounds or phases.

### 3.2. Phase Composition of the Surface Layer

**Figure 9:**
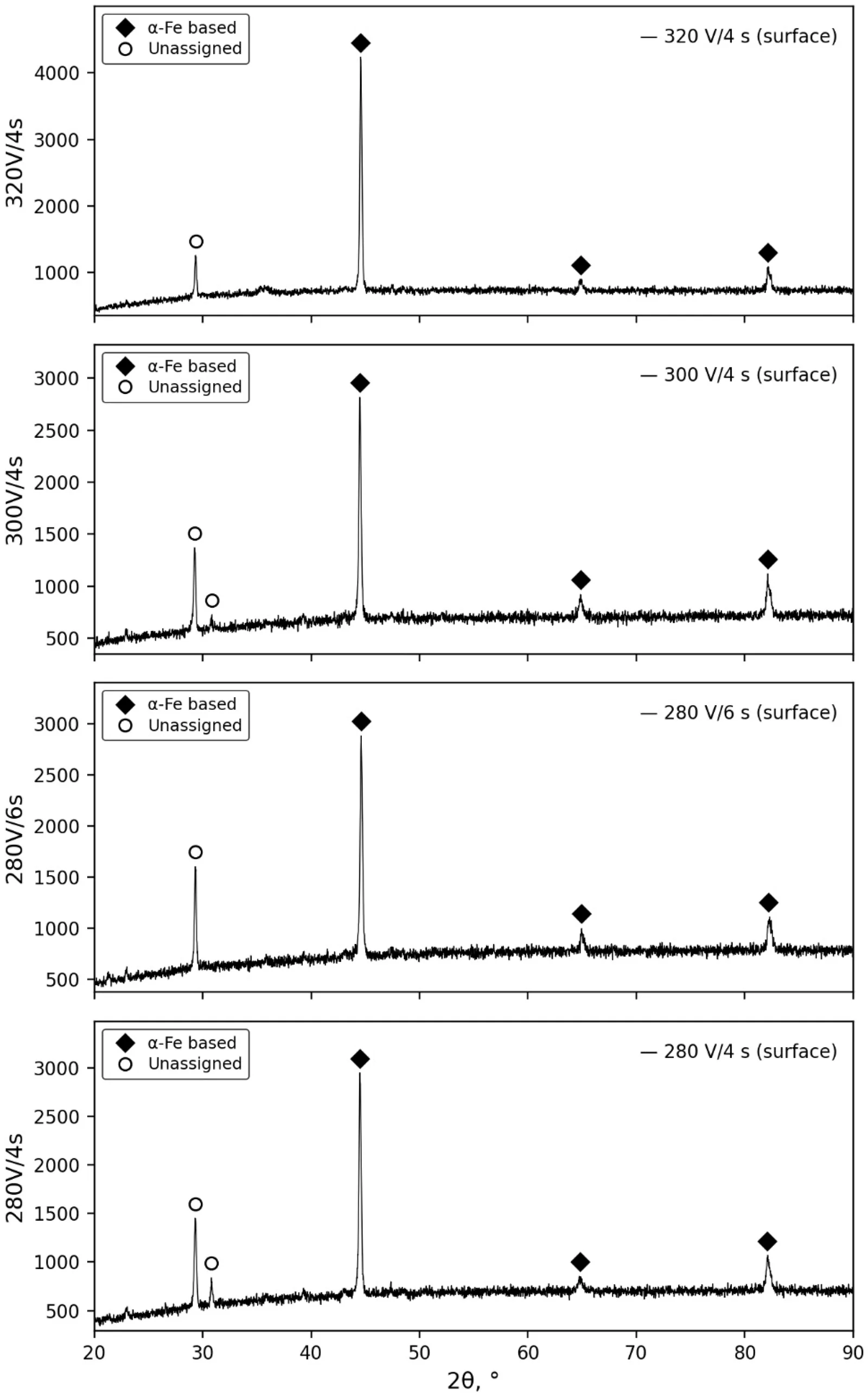
XRD patterns acquired directly from the hardened surface for 280 V/4 s, 280 V/6 s, 300 V/4 s, and 320 V/4 s samples.

**Figure 10:**
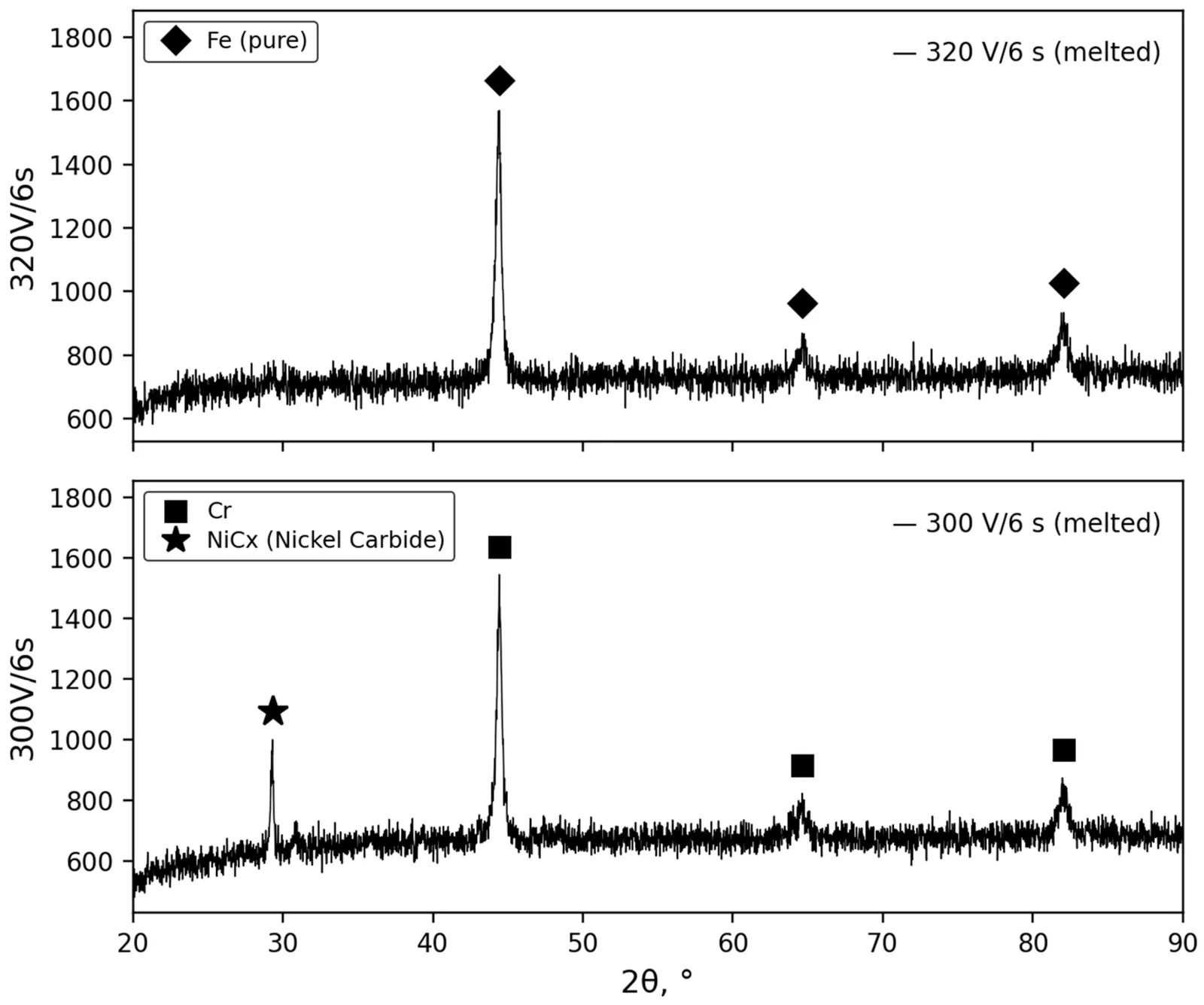
Diffractograms of 20X steel samples after 6-s EPH treatment: 300, and 320 V.

X-ray phase analysis was performed to determine the phase composition of the thermally altered layer. The diffractograms of samples treated for 4 and 6 s are shown in Figure 9 and Figure 10, respectively. The results of the identification of the main and presumed secondary phases are presented in Table 4. In the diffraction patterns of all samples studied, the main diffraction peak is located in the region of 2θ = 44.03–44.46° and corresponds to the (110) reflection of the α-Fe-based phase. In this region, the diffraction peaks of ferrite and martensite may overlap, making it difficult to unambiguously distinguish between them based on the data obtained. For this reason, the matrix component is designated in this study as an α-Fe-based phase without quantitative separation of the ferrite and martensite components. To verify the reliability of the secondary-phase identification, XRD patterns were re-acquired directly from the hardened surface (rather than from the cross-section used in the original measurements) for the 280 V/4 s, 280 V/6 s, 300 V/4 s, and 320 V/4 s conditions; the 300 V/6 s and 320 V/6 s surfaces were locally melted and could not be re-measured, so the original cross-section data are retained for these two conditions (Table 4). As can be seen in Figure 9, a weak reflection at 2θ ≈ 29.1–29.4° is reproducibly observed in all four re-measured samples alongside the main α-Fe-based peak. Automated phase search-and-match analysis (HighScore Plus) identifies the matrix reflections with an acceptable confidence (match score 53–74), whereas all candidate assignments for this secondary reflection (e.g., Fe_2_Si, SiC, Cr-Fe carbide, and elemental carbon) correspond to match scores of only 1–14, well below the level generally considered reliable (≥50). We therefore conclude that the chemical identity of this and other weak reflections cannot be established with confidence from laboratory Cu Kα XRD alone, and we report them in Table 4 without a specific phase assignment. Unambiguous identification would require further characterization (e.g., grazing-incidence XRD with extended acquisition time, or TEM/SAED), which is proposed as a direction for future work.

The diffractograms of samples treated for 6 s (Figure 10) are dominated by α-Fe-based phase reflections, whereas the number of distinct secondary peaks decreases compared to the 4-s treatment modes. No distinct secondary reflections were detected for the 280 V/6 s sample. Under the 300 V/6 s conditions, weak peaks were recorded, tentatively attributed to Cr and NiC_x_. The diffractogram of the 320 V/6 s sample is also characterized primarily by the matrix phase without clearly defined secondary peaks. The identification of the weak peaks at the 300 V/6 s setting is preliminary due to their low intensity and possible overlap of reflections. A comparison of the diffractograms shows that changing the treatment duration affects the number and intensity of the recorded secondary reflections. After a 4-s treatment, the phase composition is characterized by greater heterogeneity, whereas at a duration of 6 s, the diffractograms predominantly show an α-Fe-based phase. However, a decrease in the number of secondary peaks does not in itself indicate an improvement in the surface condition: the 280 V/6 s treatment preserves its integrity, whereas treatment at 300 and 320 V for 6 s is accompanied by localized melting. Consequently, phase changes must be considered in conjunction with the microstructural features and mechanical properties of the formed layer.

### 3.3. Mechanical Properties of the Surface Layer

**Figure 11:**
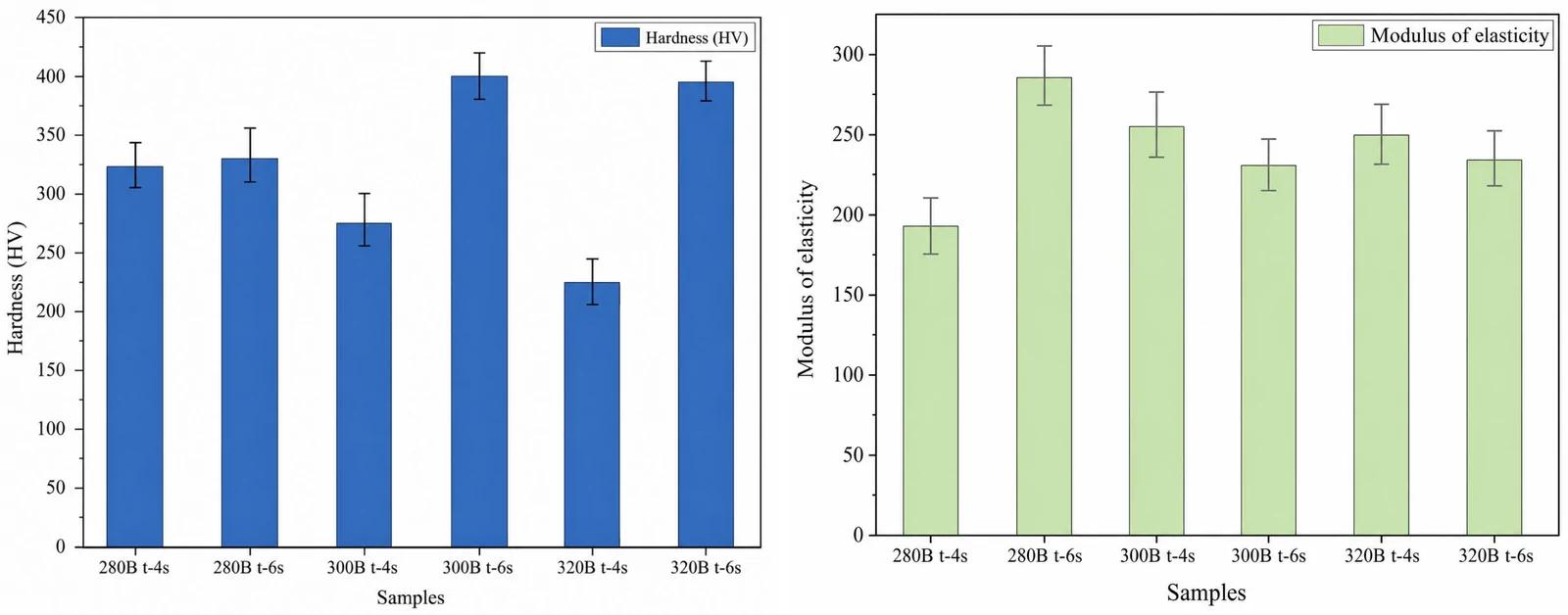
Hardness (HV) and indentation modulus (EIT) of 20X steel after electrolytic-plasma hardening under different treatment conditions.

The results of hardness and indentation modulus (EIT) measurements of the hardened layer of 20X steel after electrolytic-plasma hardening are presented in Figure 11. The hardness of the steel in its as-received condition was 180 HV.

The quantitative values of hardness and indentation modulus EIT, as well as the recorded current and surface condition of the specimens, are given in Table 5.

As shown in Figure 11 and Table 5, electrolytic-plasma hardening produced a pronounced change in the mechanical response of the surface layer. Among the specimens that retained surface integrity, the highest hardness values were obtained at 280 V/4 s and 280 V/6 s, reaching 329.6 ± 15.8 HV and 332.1 ± 26.3 HV, respectively, compared with the initial hardness of 180 HV. Thus, EPH increased the hardness by approximately 1.83–1.85 times under these conditions. Increasing the voltage from 280 to 300 and 320 V at a treatment duration of 4 s resulted in lower hardness values of 274.0 ± 13.8 HV and 225.6 ± 12.4 HV, respectively. This non-monotonic behavior indicates that the mechanical response is not controlled by the applied voltage alone, but rather by the resulting thermal cycle and the corresponding microstructural transformation of the near-surface region.

The hardness changes are consistent with the microstructural observations described in Section 3.1. EPH produced a thermally modified layer with an increasingly pronounced needle-like substructure under selected treatment conditions. Such morphology is consistent with transformation of the initially softer steel matrix toward a harder martensitic or martensite-containing microstructure during rapid heating into the austenitizing range followed by rapid cooling. However, because the diffraction reflections of ferrite and martensite overlap in the present XRD patterns, the martensite fraction cannot be quantified directly from the available XRD data. Therefore, the relationship between microstructure and hardness is interpreted based on the combined SEM, XRD, and indentation results rather than on XRD alone.

The highest hardness values, 406.2 ± 27.1 HV and 399.1 ± 28.6 HV, were obtained after treatment at 300 V/6 s and 320 V/6 s, respectively. These conditions were also characterized by a more extensively transformed needle-like microstructure, indicating a stronger thermal effect and more pronounced structural transformation. Nevertheless, localized surface melting occurred under these conditions. Therefore, although these specimens exhibited the highest hardness, these treatment parameters cannot be considered technologically optimal because the increase in hardness was accompanied by loss of surface integrity.

The indentation modulus (EIT) varied from 194.3 ± 7.4 to 286.4 ± 24.7 GPa and did not exhibit a monotonic relationship with either applied voltage or treatment duration. The lowest EIT value was obtained at 280 V/4 s, whereas the highest value was measured at 280 V/6 s. In contrast to hardness, which is strongly affected by resistance to plastic deformation and microstructural strengthening, EIT reflects the local elastic response of the indented region. Its variation may therefore be influenced by the heterogeneous phase and microstructural state of the thermally modified layer, residual stresses, and the local position of the indentation within the transformed region. Because the individual phase fractions were not quantitatively determined in the present study, the EIT values cannot be assigned directly to a specific ferritic, pearlitic, or martensitic phase. Nevertheless, the observed variation in EIT confirms that EPH modifies not only the hardness but also the local elastic response of the surface layer.

As shown in Table 5, the recorded current exhibited a non-monotonic dependence on the treatment conditions. Thus, the measured current alone does not provide a sufficient basis for assessing the intensity or uniformity of local thermal exposure during electrolytic-plasma hardening. The observed variations in current indicate that the electrical response of the system depends on the treatment conditions in a non-monotonic manner. However, the specific mechanism responsible for these variations and for the localized melting observed at 300 V/6 s and 320 V/6 s cannot be established from the present measurements alone. In particular, the available data do not allow us to directly attribute this behavior to instability of the vapor–gas envelope or to a specific pattern of energy redistribution. Resolving this issue would require time-resolved monitoring of current and voltage during treatment, together with direct temperature measurements or other in situ diagnostics.

**Figure 12:**
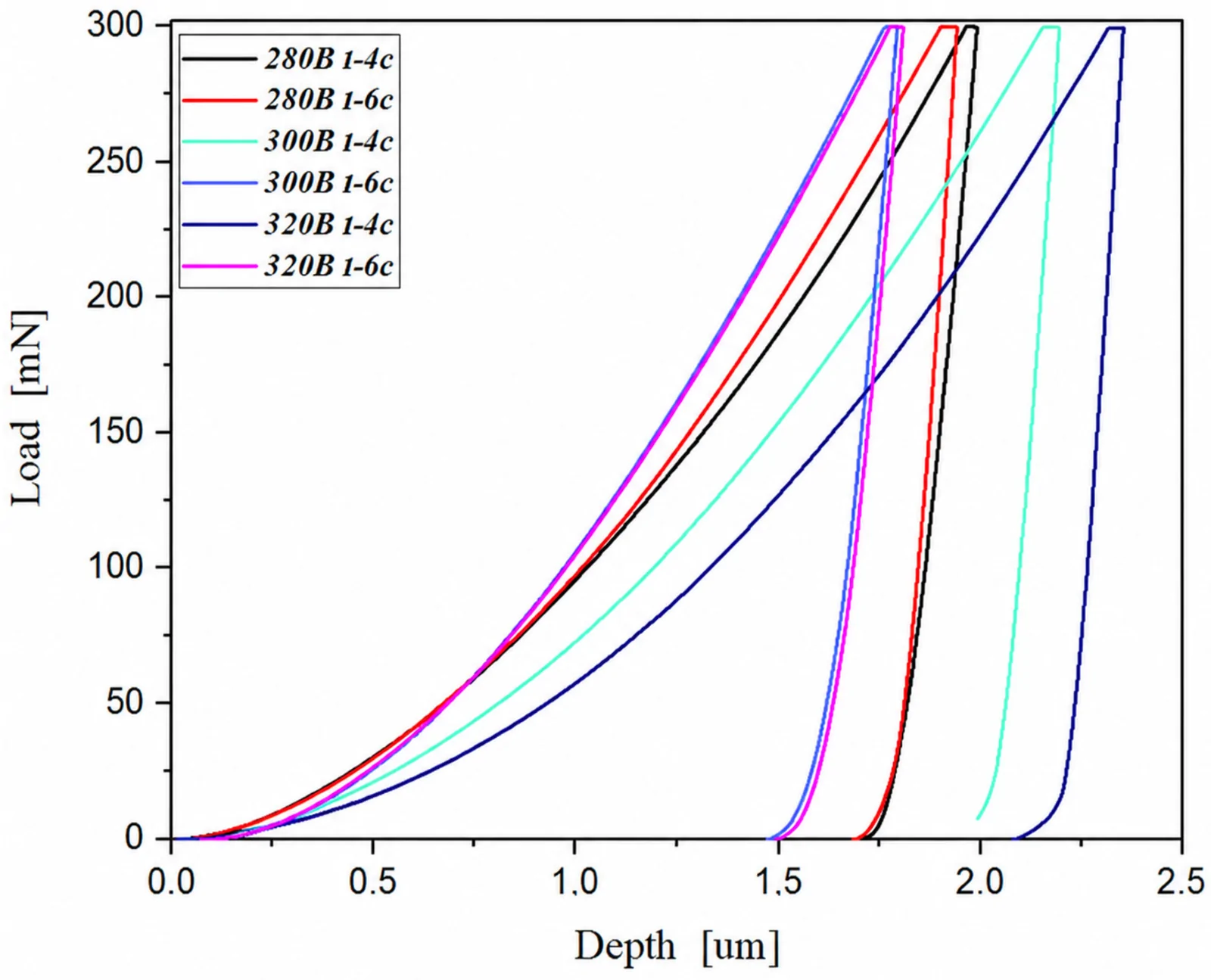
“Load–Depth” curves from instrumental indentation tests on 20X steel specimens after electrolytic-plasma hardening under various processing conditions.

The load–depth curves obtained by instrumented indentation are presented in Figure 12. At the same maximum load of 300 mN, a lower penetration depth corresponds to a higher resistance to indentation. The specimens treated at 300 V/6 s and 320 V/6 s exhibited a maximum penetration depth of approximately 1.75 μm, whereas the 320 V/4 s specimen showed a penetration depth of approximately 2.3 μm. These differences are consistent with the hardness values presented in Table 5 and further confirm the effect of the EPH-induced microstructural changes on the mechanical response of the treated surface layer.

The maximum hardness achieved in this study among undamaged specimens (332.1 HV at 280 V/6 s) is considerably lower than the surface hardness typically reported for carburized and quenched low-alloy case-hardening steels closely related in composition to 20X steel. For low-alloy chromium–nickel gear steels such as 18Cr2Ni4WA and similar grades, carburizing–quenching(–tempering) treatments typically produce a surface hardness in the range of approximately 545–820 HV, depending on the carburizing time and subsequent tempering conditions [20,21]. This comparison confirms that, in terms of absolute surface hardness, the EPH conditions investigated in the present study do not reach the hardness level typically attainable by conventional carburizing followed by quenching. Therefore, the principal advantage of EPH in the present context is not the achievement of maximum surface hardness, but the possibility of rapid and localized surface modification within a treatment time of only a few seconds. Accordingly, the practical value of the method examined in the present study should not be interpreted as a direct substitute for carburizing in applications where maximum case hardness is the governing requirement, but rather as a complementary technology suited to cases in which localized surface heating, preservation of the core microstructure, and the absence of furnace or vacuum equipment are prioritized over achieving the maximum possible surface hardness. This may be particularly relevant for small-batch and repair production, as well as for components where minimizing thermal distortion is important.

### 3.4. Tribological Properties

**Figure 13:**
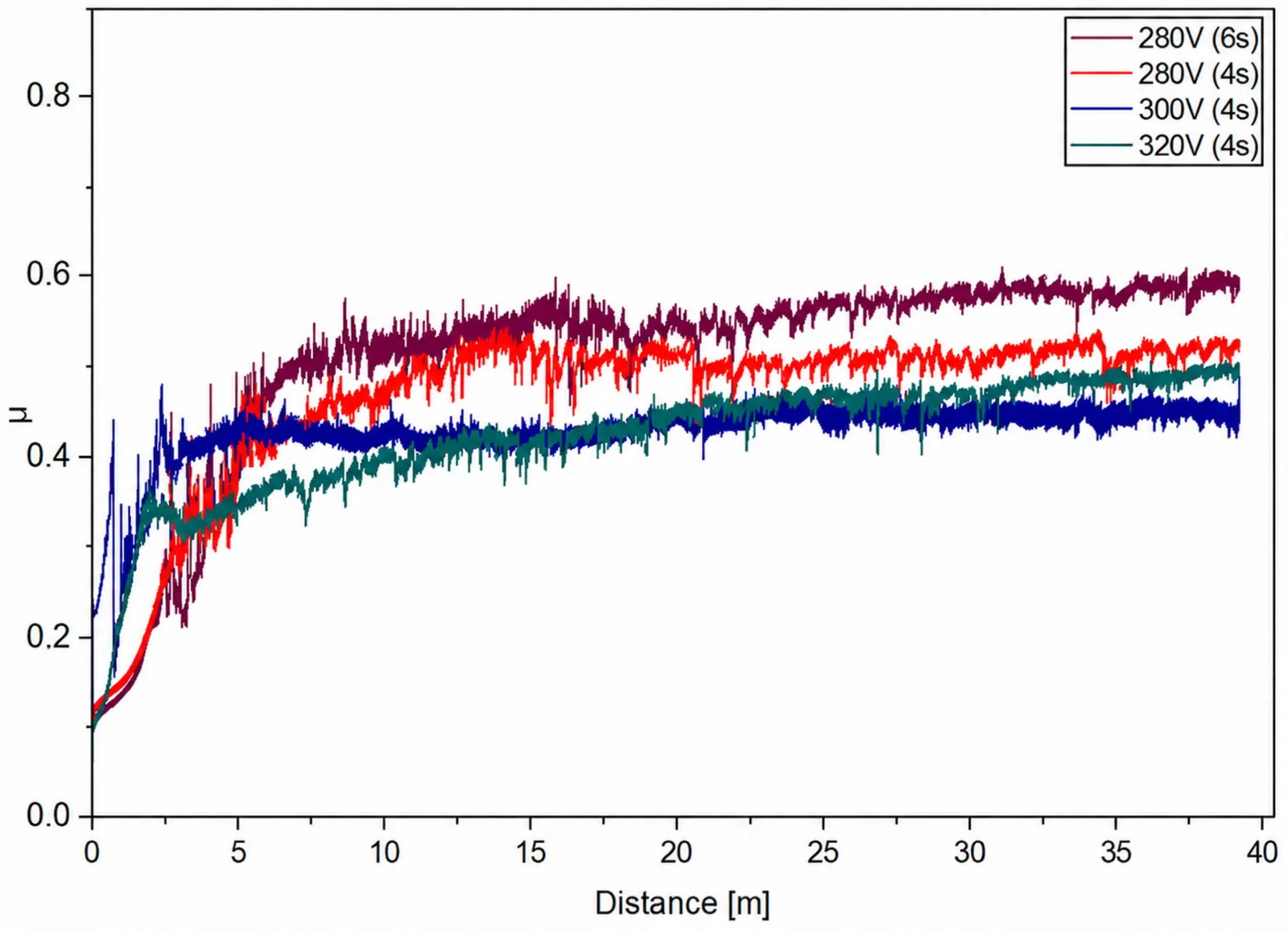
Dependence of the coefficient of friction μ on friction distance for 20X steel specimens after electrolytic-plasma hardening under the following conditions: 280 V/4 s, 300 V/4 s, 320 V/4 s, and 280 V/6 s.

To evaluate the effect of the formed microstructure and hardness of the surface layer on its performance characteristics, tribological tests were conducted on samples that retained surface integrity after electrolytic-plasma hardening. The relationships between the coefficient of friction μ and the friction distance for the 280 V/4 s, 300 V/4 s, 320 V/4 s, and 280 V/6 s samples are shown in Figure 13. The 300 V/6 s and 320 V/6 s samples were not tested due to surface melting.

The values of the coefficient of friction during the running-in phase (0–5 m) and the steady-state friction phase (>5 m) are given in Table 6.

In the steady-state phase, the minimum average coefficient of friction was recorded for the 300 V/4 s sample and amounted to 0.444 ± 0.054. For the 320 V/4 s and 280 V/4 s modes, the coefficient of friction values were 0.464 ± 0.055 and 0.508 ± 0.044, respectively. The maximum value, equal to 0.572 ± 0.058, was observed for the 280 V/6 s sample. A comparison of the obtained results with indentation data did not reveal a clear dependence of the coefficient of friction on hardness. In particular, the 280 V/4 s and 280 V/6 s samples had similar hardness values—329.6 and 332.1 HV, respectively—but differed in their coefficient of friction. Furthermore, the 300 V/4 s sample, with a hardness of 274.0 HV, exhibited a lower coefficient of friction than the less hard 320 V/4 s sample. This indicates that tribological behavior was determined not only by hardness but also by the combined influence of microstructure, phase composition, and the condition of the contact surface. In particular, at 280 V the extension of treatment duration from 4 to 6 s was accompanied by an intensification of the needle-like substructure and by the formation of more sharply defined internal boundaries within the thermally altered layer (Section 3.1), while the average hardness remained essentially unchanged (329.6 vs. 332.1 HV). The higher surface roughness of the 280 V/6 s specimen, together with the more pronounced structural heterogeneity observed in the thermally modified layer, may contribute to its higher coefficient of friction and wider wear track compared with the 280 V/4 s condition. However, the present results do not allow the individual contributions of surface roughness, microstructure, and phase state to the tribological response to be quantitatively separated. In addition, the weak secondary reflections identified by XRD differed between conditions (Table 4): candidate assignments included Fe_2_Si and elemental carbon at 280 V/4 s, Cr-Fe carbide and carbon at 300 V/4 s, and carbon alone at 320 V/4 s, although none of these assignments could be established with confidence (match scores ≤ 14).

**Figure 14:**
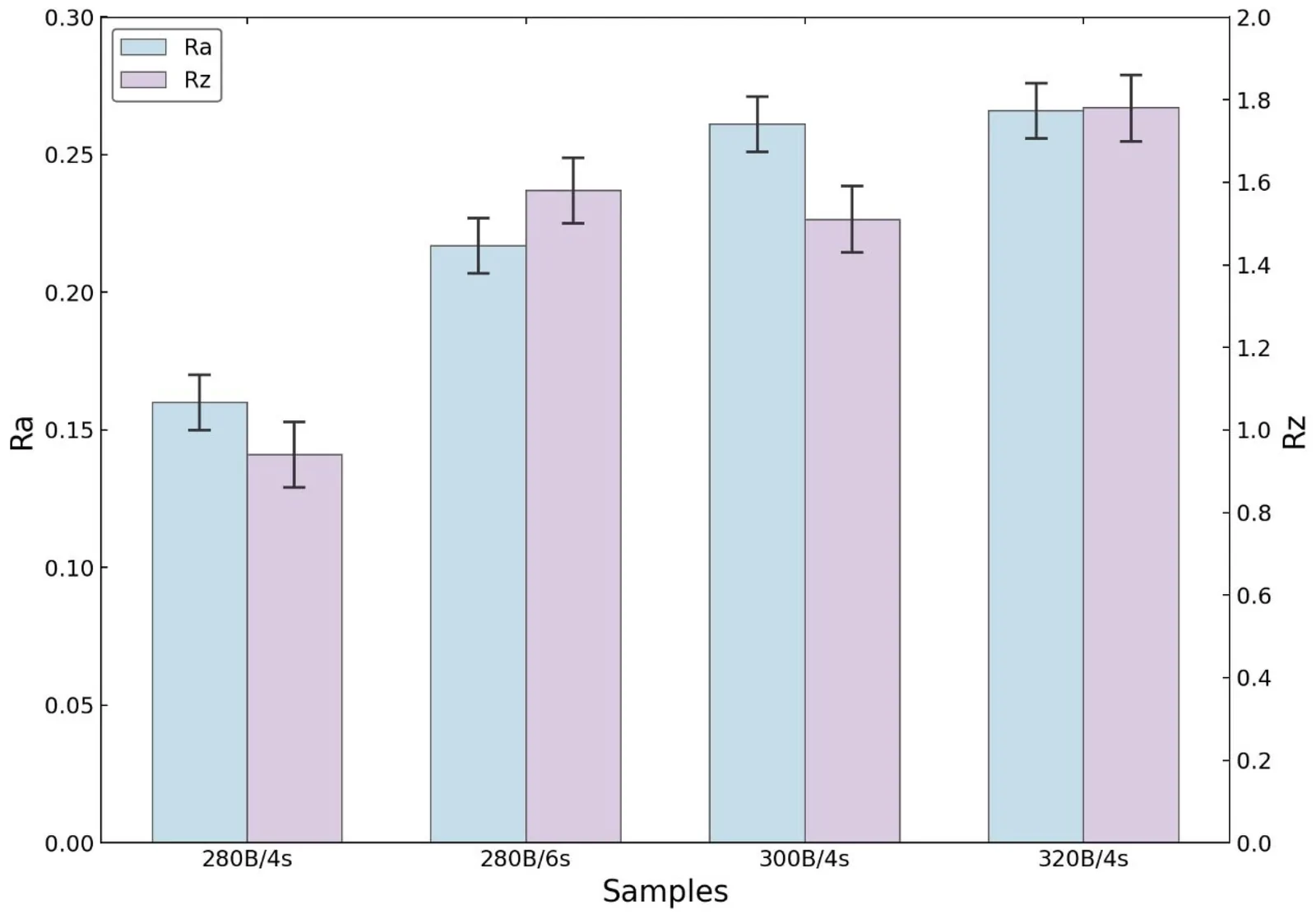
Surface roughness (Ra) of 20X steel after electrolytic-plasma hardening under different treatment conditions.

Surface roughness was additionally characterized by profilometry to evaluate the possible contribution of surface topography to the observed tribological differences. The average Ra values were 0.160 ± 0.008 μm for 280 V/4 s, 0.217 ± 0.005 μm for 280 V/6 s, 0.262 ± 0.015 μm for 300 V/4 s, and 0.267 ± 0.050 μm for 320 V/4 s. The corresponding results are presented in Figure 14.

Notably, the 280 V/4 s and 280 V/6 s samples exhibited nearly identical hardness values of 329.6 and 332.1 HV, respectively, whereas their surface roughness values differed. The higher Ra value for the 280 V/6 s condition may contribute to its higher coefficient of friction and wider wear track compared with the 280 V/4 s condition. However, surface roughness alone cannot fully account for the observed tribological differences, since microstructural features and surface phase composition may also influence the friction and wear behavior.

**Figure 15:**
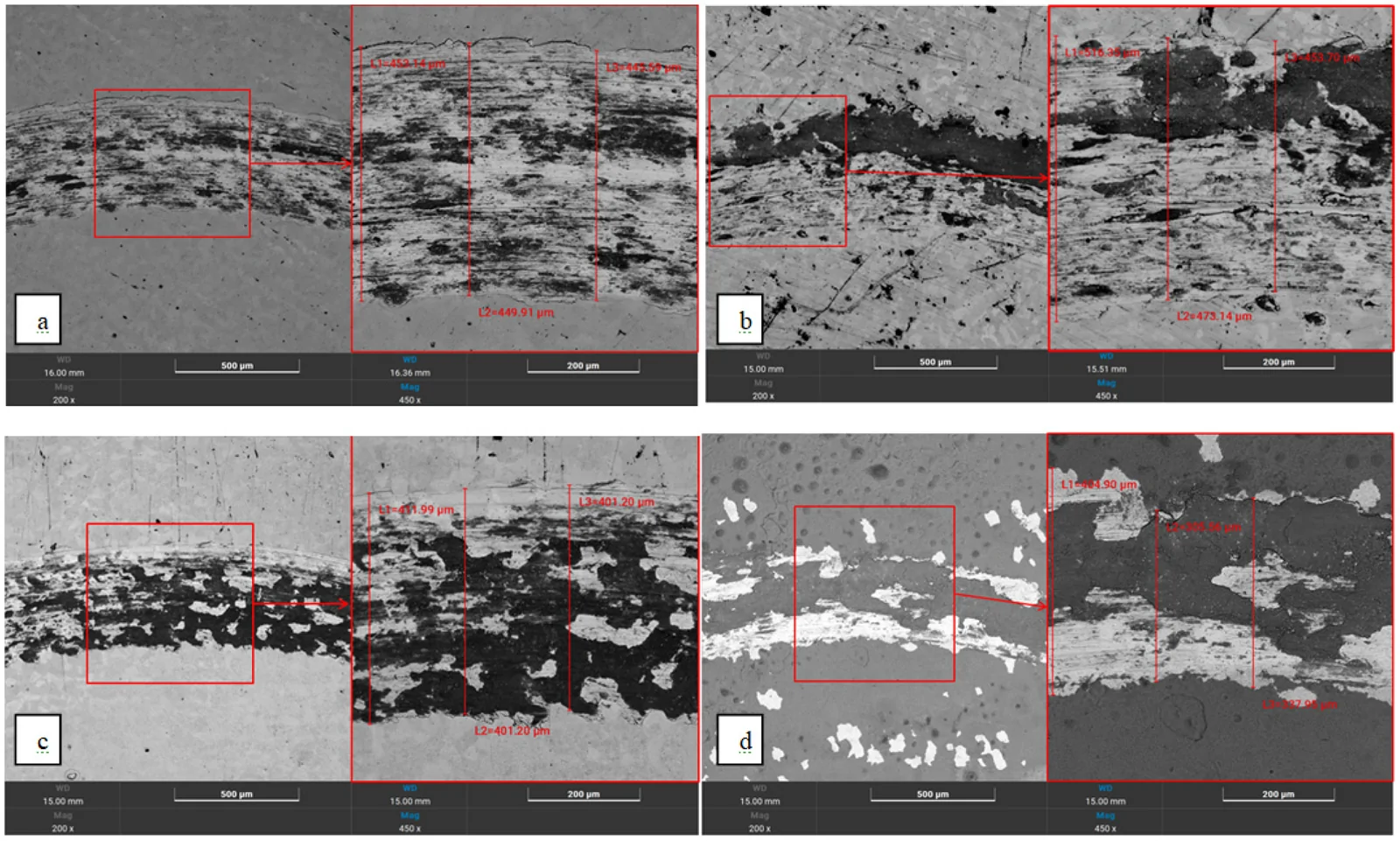
SEM images of wear tracks on the surface of 20X steel after electrolytic-plasma hardening: (**a**) 280 V/4 s; (**b**) 280 V/6 s; (**c**) 300 V/4 s; (**d**) 320 V/4 s.

To comparatively evaluate wear after tribological testing, the morphology of the wear tracks was examined using SEM, and their width was measured at three points on each specimen. SEM images of the wear tracks are shown in Figure 15, and the measurement results are presented in Table 7.

The minimum average wear track width was recorded for the 320 V/4 s sample and amounted to 379.48 ± 36.24 μm. For the 300 V/4 s and 280 V/4 s samples, the wear track width increased to 404.80 ± 6.23 and 448.55 ± 5.41 μm, respectively. The maximum value, equal to 481.07 ± 32.07 μm, was obtained at the 280 V/6 s operating mode.

A comparison of the results showed that the increase in hardness within the studied range was not accompanied by a decrease in the width of the wear track. In particular, the 280 V/4 s and 280 V/6 s samples exhibited the highest hardness among the undamaged samples but were characterized by wider wear tracks and higher coefficient of friction values. When the treatment duration at 280 V was increased from 4 to 6 s, the hardness remained virtually unchanged, whereas the coefficient of friction increased from 0.508 to 0.572, and the wear track width increased from 448.55 to 481.07 μm.

The results obtained indicate that there is no clear correlation between tribological behavior and hardness of the treated layer alone. It is likely that the microstructure, phase composition, and surface condition—formed under various electrolytic-plasma hardening conditions—had an additional influence. However, the track width characterizes only the geometry of the damaged area and does not allow for a direct determination of the wear volume or wear rate. Therefore, the obtained values should be used for a comparative evaluation of the investigated modes.

For future optimization of electrolytic-plasma hardening, data-driven and interpretable machine-learning approaches could be used to establish relationships between processing parameters (voltage and treatment time) and the resulting microstructural, hardness, and tribological characteristics. Such approaches may provide a systematic framework for optimizing multiple process parameters and identifying their relative influence on the surface properties [22,23,24,25,26]. However, the present dataset includes only six processing conditions and is therefore insufficient for the development of a statistically reliable machine-learning model; such an analysis would require a substantially expanded experimental dataset covering a broader processing-parameter space.

## 4. Conclusions

The effects of applied voltage and treatment duration on the surface modification of 20X steel by electrolytic-plasma hardening were systematically investigated. The 280 V/4 s, 300 V/4 s, 320 V/4 s, and 280 V/6 s conditions produced thermally modified surface layers without visible surface damage, whereas increasing the treatment duration to 6 s at 300 and 320 V resulted in localized surface melting and therefore exceeded the technologically stable EPH range.

Among the undamaged specimens, the highest hardness was obtained at 280 V, reaching 329.6 ± 15.8 HV after 4 s and 332.1 ± 26.3 HV after 6 s, compared with an initial hardness of 180 HV. Thus, EPH increased the hardness by approximately 1.83–1.85 times under these conditions. Although higher hardness values of 406.2 ± 27.1 HV and 399.1 ± 28.6 HV were obtained at 300 V/6 s and 320 V/6 s, respectively, these regimes cannot be considered technologically acceptable because of localized surface melting.

XRD analysis showed that the treated layers were dominated by an α-Fe-based phase. Weak additional reflections were observed under several treatment conditions; however, their phase assignment could not be established reliably from the available laboratory XRD data. The structural changes observed by SEM therefore should be interpreted together with the XRD and mechanical-property results rather than attributed to specific phases on the basis of weak diffraction reflections alone.

The tribological response did not correlate directly with hardness. The lowest steady-state coefficient of friction, 0.444 ± 0.054, was obtained at 300 V/4 s, whereas the narrowest wear track, 379.48 ± 36.24 μm, was recorded at 320 V/4 s. In contrast, the 280 V/4 s and 280 V/6 s specimens exhibited the highest hardness among the undamaged samples but also showed higher coefficients of friction and wider wear tracks. These results indicate that hardness alone is insufficient for predicting tribological performance and that surface roughness and microstructural state must also be considered.

Overall, the 280 V/4 s condition provides the most favorable combination when increased hardness, preservation of surface integrity, and minimum treatment duration are prioritized. The 300 V/4 s and 320 V/4 s conditions may be preferable when the primary criterion is a lower coefficient of friction or a narrower wear track, respectively. The results demonstrate that EPH should be considered as a rapid and localized surface-hardening approach rather than a direct replacement for conventional carburizing when maximum surface hardness is required.

## Figures and Tables

**Table 1:** Chemical composition of 20X steel (GOST 4543-2016) [9]

C	Si	Mn	Cr	Ni	S	P	Cu	Fe
0.17–0.23	0.17–0.37	0.50–0.80	0.70–1.00	≤0.30	≤0.035	≤0.035	≤0.30	Base

**Table 2:** Electrolytic-plasma hardening conditions for 20X steel samples.

No.	Voltage, V	Time, s	Current, A
1	280	4	14.2
2	300	4	14.6
3	320	4	12.6
4	280	6	29.2
5	300	6	12.7
6	320	6	23.6

**Table 3:** Semi-quantitative elemental composition of the samples based on linear EMF scanning data, wt.%.

Sample No.	Test Conditions	Fe	C	O	Cr	Mn	Si
1	280 V/4 s	90.3	7.7	—	0.9	0.8	0.3
2	280 V/6 s	89.4	9.1	—	0.9	0.6	—
3	300 V/4 s	89.4	8.3	—	0.9	1.0	0.3
4	320 V/4 s	88.77	8.52	0.99	0.84	0.64	0.25
5	300 V/6 s (fused)	86.20	11.23	1.62	0.65	—	0.31
6	320 V/6 s (melted)	84.04	13.07	2.33	0.57	—	—

**Table 4:** Phase composition of the surface layer of 20X steel after electrolytic plasma hardening based on XRD data. Data for 280 V/4 s, 280 V/6 s, 300 V/4 s, and 320 V/4 s were re-acquired directly from the hardened surface; * data for 300 V/6 s and 320 V/6 s are from the original cross-section measurements, as the melted surface of these samples could not be re-measured.

Treatment Mode	2θ of the Main Peak, °	Matrix Phase	Presumed Secondary Phases
280 V/4 s (surface)	44.49	α-Fe based (score 53)	Weak reflection at 2θ ≈ 29.3°; phase assignment not reliable (best candidate score ≤ 14)
300 V/4 s (surface)	44.48	α-Fe based (score 63)	Weak reflection at 2θ ≈ 29.3°; phase assignment not reliable (best candidate score ≤ 10)
320 V/4 s (surface)	44.54	α-Fe based (score 55)	Weak reflection at 2θ ≈ 29.4°; phase assignment not reliable (best candidate score 11)
280 V/6 s (surface)	44.60	α-Fe based (score 74)	Weak reflection at 2θ ≈ 29.3°; phase assignment not reliable (best candidate score ≤ 10)
300 V/6 s (cross-section) *	44.42	α-Fe(Cr)	Weak reflections detected; phase assignment remains tentative because of their low intensity and overlap
320 V/6 s (cross-section) *	44.44	α-Fe(Cr)	No secondary phases detected

**Table 5:** Mechanical properties and surface condition of 20X steel after electrolytic-plasma hardening.

Mode	I, A	Hardness, HV	EIT, GPa	Surface Condition
280 V/4 s	14.2	329.6 ± 15.8	194.3 ± 7.4	No visible damage
300 V/4 s	14.6	274.0 ± 13.8	254.5 ± 23.5	No visible damage
320 V/4 s	12.6	225.6 ± 12.4	250.0 ± 16.7	No visible damage
280 V/6 s	29.2	332.1 ± 26.3	286.4 ± 24.7	No visible damage
300 V/6 s	12.7	406.2 ± 27.1	232.0 ± 22.8	Surface melting
320 V/6 s	23.6	399.1 ± 28.6	232.5 ± 20.9	Surface melting

**Table 6:** Coefficient of friction for 20X steel samples in the running-in and steady-state friction sections.

Processing Mode	Hardness, HV	μ, Running-in Section 0–5 m	μ, Steady-State Friction Section >5 m
280 V/4 s	329.6	0.248	0.508 ± 0.044
300 V/4 s	274.0	0.363	0.444 ± 0.054
320 V/4 s	225.6	0.288	0.464 ± 0.055
280 V/6 s	332.1	0.240	0.572 ± 0.058

**Table 7:** Average width of wear tracks in 20X steel samples after electrolytic-plasma hardening.

Treatment Mode	Hardness, HV	Coefficient of Friction, μ	Wear Track Width, μm
280 V/4 s	329.6	0.508	448.55 ± 5.41
300 V/4 s	274.0	0.444	404.80 ± 6.23
320 V/4 s	225.6	0.464	379.48 ± 36.24
280 V/6 s	332.1	0.572	481.07 ± 32.07

## Data Availability

The original contributions presented in this study are included in the article. Further inquiries can be directed to the corresponding author.
